# Ecdysone promotes growth of imaginal discs through the regulation of Thor in *D. melanogaster*

**DOI:** 10.1038/srep12383

**Published:** 2015-07-22

**Authors:** Leire Herboso, Marisa M. Oliveira, Ana Talamillo, Coralia Pérez, Monika González, David Martín, James D. Sutherland, Alexander W. Shingleton, Christen K. Mirth, Rosa Barrio

**Affiliations:** 1 CIC bioGUNE, Bizkaia Technology Park, 48160 Derio, Bizkaia, Spain; 2 Instituto Gulbenkian de Ciência, Rua da Quinta Grande, 6, 2780-156 Oeiras, Portugal; 3 Institute of Evolutionary Biology, CSIC–Universitat Pompeu Fabra, 08003 Barcelona, Spain; 4 Lake Forest College, 555 North Sheridan Road, Lake Forest, IL 60045, USA

## Abstract

Animals have a determined species-specific body size that results from the combined action of hormones and signaling pathways regulating growth rate and duration. In *Drosophila*, the steroid hormone ecdysone controls developmental transitions, thereby regulating the duration of the growth period. Here we show that ecdysone promotes the growth of imaginal discs in mid-third instar larvae, since imaginal discs from larvae with reduced or no ecdysone synthesis are smaller than wild type due to smaller and fewer cells. We show that insulin-like peptides are produced and secreted normally in larvae with reduced ecdysone synthesis, and upstream components of insulin/insulin-like signaling are activated in their discs. Instead, ecdysone appears to regulate the growth of imaginal discs via *Thor/4E-BP*, a negative growth regulator downstream of the insulin/insulin-like growth factor/Tor pathways. Discs from larvae with reduced ecdysone synthesis have elevated levels of *Thor*, while mutations in *Thor* partially rescue their growth. The regulation of organ growth by ecdysone is evolutionarily conserved in hemimetabolous insects, as shown by our results obtained using *Blattella germanica*. In summary, our data provide new insights into the relationship between components of the insulin/insulin-like/Tor and ecdysone pathways in the control of organ growth.

Animal size is species-specific. The final size of an organ, and that of the whole body, is determined by the size and number of its cells. To reach adult size, animals must control i) the growth rate and ii) the duration of the growth period. These parameters are regulated by hormones and the insulin/insulin-like growth factor (IIS) and Target of Rapamycin (Tor) pathways (IIS/Tor)^1^,^2^. In flies and mice, IIS/Tor pathways control rates of cell growth, nutrient use and cell and body size, while steroid hormones regulate developmental transitions^3^. The insect steroid hormone 20-hydroxyecdysone (20E, referred to here as ecdysone) also exerts a negative role in systemic growth^4^ and promotes organ differentiation at the end of the larval period.

In holometabolous insects, adult body size is defined by the final larval size. *Drosophila* passes through three larval stages (L1-L3) where larval tissues undergo an impressive increase in mass, a process regulated by environmental signals (temperature, oxygen, nutrition), as well as internal signals (hormones)^5^,^6^. Much of the increase in tissue mass is a consequence of IIS/Tor pathways. In larval fat body (FB), a tissue in flies which is homologous to vertebrate liver and adipose tissues, Tor is activated by amino acids^7^ and promotes the release of a FB secreted signal, which controls the secretion of insulin-like peptides (dIlps) from the brain^8^. Dietary sugar and fat promote the secretion of Unpaired 2, equivalent to human Leptin, which also promotes dIlp secretion^9^. dIlps induce cell and tissue growth, linking nutrition to systemic growth. There are 8 dIlps in *Drosophila*, closely related to human insulin^10^,^11^,^12^. dIlp2, 3 and 5 are synthesized in brain neurosecretory insulin producing cells (IPCs), which are functionally similar to the pancreatic β-cells^10^,^13^. IPC ablation produces a phenotype of small, normally-proportioned adults by reducing cell size and number^13^. Starvation also stops growth, as under nutrient deprivation dIlps are no longer secreted and accumulate in the IPCs^8^,^9^.

Ecdysone controls molting and the onset of metamorphosis in insects^14^,^15^. The precursor of ecdysone is produced from cholesterol in the prothoracic glands (PG), and is modified into its active form in peripheral tissues^16^. Ecdysone activates stage-specific gene cascades that determine the timing of developmental progression^15^. In the last larval instar, high levels of ecdysone promote the transition to pupal development and reduce growth via the FB. There, it represses the expression of Diminutive (Dm, also known as Myc), which induces the production of an unknown endocrine signal that in turn promotes systemic growth^4^,^17^. Thus, alterations in the production of ecdysone affect final animal size. For instance, activation of IIS/Tor signaling in the PG increases ecdysone levels and causes reduced growth rates and precocious pupariation, resulting in animals that are smaller than wild type (WT)^18^,^19^.

Silencing the *Drosophila Small Ubiquitin Modifier* (*SUMO) 2/3* homologue, *smt3*, in the PG (*smt3i*) prevents larvae from entering pupariation, arresting their development at the third larval instar (L3) due to their inability to synthesize high levels of ecdysone^20^,^21^. These larvae continue feeding and growing for several weeks, giving rise to big L3. We hypothesized that larval organs might grow proportionally to the body of *smt3i* larvae. However, while the gut of these larvae grew proportionally with body size, we discovered that the imaginal tissues did not. We found that ecdysone was necessary for cell growth and proliferation in mid-late L3 larvae, and exerted its regulation on growth via Thor, the *Drosophila* 4E-BP homolog, which may affect the IIS/Tor pathways. Therefore, ecdysone plays a complex and conflicting role in growth regulation, inhibiting systemic growth of larval tissues through the repression of Dm in the FB^4^ while inducing growth of the imaginal tissues.

## Results

### Reduced levels of ecdysone impair organ growth

**Figure 1:**
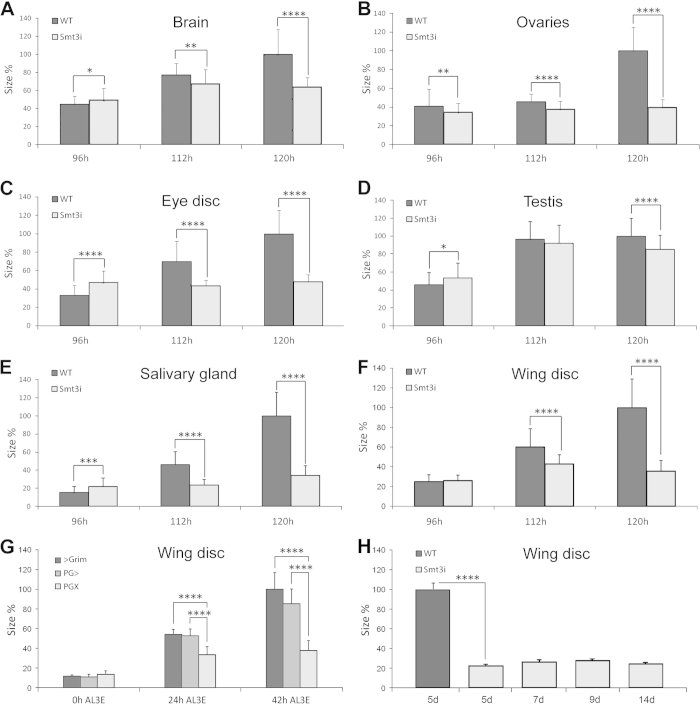
Organ size depends on ecdysone. (**A**–**F**,**H**) Average size of WT or *smt3i* organs at the indicated times. (**G**) Wing disc size of *Grim >*, *PG > *or *PGX* larvae at 0, 24 and 42 hours AL3E. Error bars indicate standard deviation. Measurements are expressed in percentage relative to control organs at 120 hours AEL (**A**–**F**,**H**) or 42 hours AL3E (**G**). One asterisk indicates p ≤ 0.05, two p ≤ 0.01, three p ≤ 0.001 and four p ≤ 0.0001. d: days.

Organs of *smt3i* L3 larvae are smaller than those from controls (Fig. 1A–F). To understand when the growth of the organs is impaired, we analyzed three different time points: mid L3 96 hours after egg laying (AEL), early-wandering 112 hours AEL and late-wandering 120 hours AEL L3 larvae. Control organs grow from 96 to 120 hours AEL (Fig. 1A–F), except for the testis. However, *smt3i* organs stop their growth between 96 and 112 hours AEL. At 96 hours AEL, *smt3i* organs are moderately bigger than controls, except for the ovaries. At 112 hours AEL most organs are significantly smaller in *smt3i* compared to controls (Fig. 1A–F). This difference in size increases with time, as control organs continue growing while organs from *smt3i* larvae do not change significantly (Fig. 1). Importantly, wing discs do not grow significantly after several weeks of *smt3i* larval life (Fig. 1H), indicating a developmental arrest between 96 and 112 hours AEL rather than a delay in growth. In conclusion, reduced levels of ecdysone during L3 impair the growth of imaginal organs in mid to late L3. Remarkably, larval gut is bigger than control (Supplementary Fig. S1) and could partially account for the increase in body mass reported for the *smt3i* larvae^20^.

To confirm that the suppressed growth was due to reduced levels of ecdysone, we used a second method to decrease ecdysone titers by genetically ablating the PG at L3 (Supplementary Fig. S2). *phm* >* grim, tub-Gal80*^*ts*^ larvae reared at 17 °C develop normally (Supplementary Fig. S2). However, larvae shifted to 29 °C at the beginning of L3 (called *PGX* larvae) do not pupariate, continue to feed and exhibit a highly reduced and deformed ring gland complex at 42 hours after ecdysis to the third instar (AL3E; Supplementary Fig. S3). *PGX* and control larvae increase equally in weight from 0 to 24 hours AL3E (Supplementary Fig. S3). Between 24 and 42 hours AL3E, however, *PGX* larvae show a greater increase in weight than the controls (Supplementary Fig. S3). While the control larvae wander then pupariate between 42 and 45 hours, *PGX* larvae continue to feed and increase in weight for weeks (Supplementary Fig. S3). Thus, like *smt3i*, *PGX* larvae show developmental arrest and increased body size. A schematic equivalence of the time scale between the two systems is included in Supplementary Fig. S2D.

Discs from *PGX* larvae are smaller than those from controls (Fig. 1G). At the molt to L3, no differences in disc size were observed, but at 24 hours AL3E, *PGX* discs exhibit lower growth rates than both controls (Fig. 1G). Overall, this suggests that the growth of the wing discs from *PGX* larvae is dramatically impaired. Furthermore, it corroborates the *smt3i* results, showing that ecdysone is necessary for the growth of the imaginal tissues. Due to the similarity in phenotype between *smt3i* and *PGX* larvae, we conducted most of the following experiments in *smt3i* larvae.

To further support our hypothesis that the lack of growth is due to the direct effect of ecdysone on the imaginal tissue, we overexpressed a dominant negative transgene of EcR, *UAS-EcR B1*^*w650*^, using the *C765-Gal4* driver. Whereas the overexpression of this transgene does not have any effect at 7 h AL3E, it causes a reduction of the wing imaginal disc size compared to the control at 31h AL3E (Supplementary Fig. S4). These results are compatible with a direct effect of ecdysone on the imaginal tissues in mid-late L3 larvae.

### Expression of ecdysone-inducible genes is altered in discs from *smt3i* larvae

Our results suggest that discs grow in response to ecdysone during the L3 instar larvae. Therefore, ecdysone-dependent genes might display altered expression in *smt3i* larvae. To address this, we focused on the wing discs, which have been extensively used as a paradigm to study the roles of signaling pathways in organ growth.

**Figure 2:**
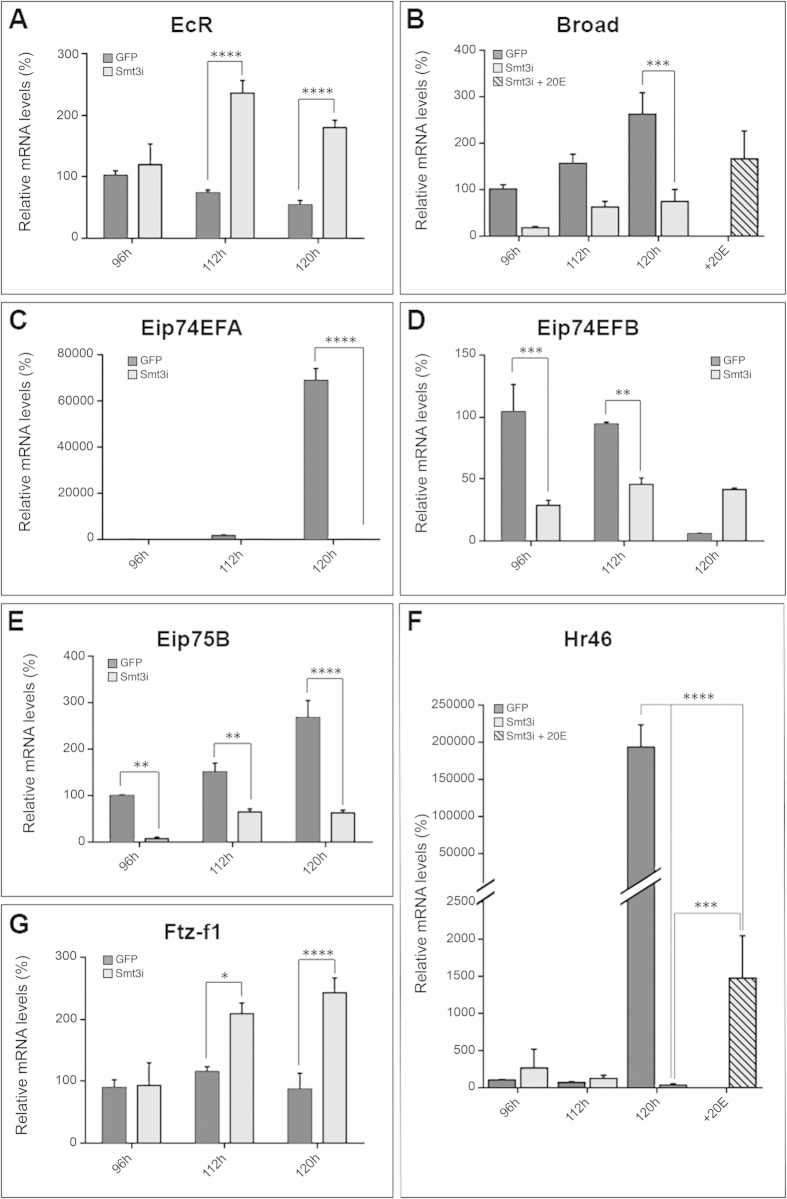
Gene expression regulation is altered in wing discs from low-ecdysone larvae. (**A**–**F**) qPCR analysis of the indicated genes using mRNA from wing discs of *GFP* or *smt3i* larvae fed with food supplemented or not with ecdysone (+20E). Error bars indicate standard deviation. Values are expressed in percentage relative to 96 hours AEL control discs. One asterisk indicates p ≤ 0.05, two p ≤ 0.01, three p ≤ 0.001, and four p ≤ 0.0001.

Our qPCR analysis showed that the expression of ecdysone-response genes in the wing discs of *smt3i* larvae is consistent with a systemic suppression of ecdysone signaling. We examined several ecdysone-inducible genes [*broad* (*br*), *Ecdysone-induced protein 74EF* (*Eip74EFA*), *Ecdysone-induced protein 75B* (*Eip75B*) and *Hormone receptor-like in 46* (*Hr46*, also known as *DHR3*)], as well as ecdysone-repressed genes [*Ecdysone Receptor* (*EcR*), *Eip74EFB* and *fushi tarazu factor 1* (*ftz-f1*)]^22^,^23^,^24^,^25^,^26^. Our results showed that genes normally activated by ecdysone are upregulated in discs from control *GFP* larvae at 112 or 120 hours AEL, compared to 96 hours AEL control discs or *smt3i* discs of equivalent age (Fig. 2B,C,E,F). Conversely, genes normally repressed by ecdysone are downregulated in discs from control *GFP* larvae at 120 hours AEL compared to *smt3i* discs of equivalent age (Fig. 2A,D,G). Collectively, these results show that ecdysone signaling is active in control *GFP* discs, but it is suppressed in *smt3i* discs. Consistent with this hypothesis, exogenous ecdysone administration increases the levels of *br* and *Hr46* in *smt3i* discs (Fig. 2B,F).

Ecdysone also regulates early stages of patterning in the wing disc^27^. To assess if patterning is affected we used immunocytochemistry to characterize the expression of Cut (Ct) and Wingless (Wg) in control and *smt3i* discs. The expression pattern of Wg and Ct in *smt3i* discs at 120 hours AEL is similar to their expression in WT discs at earlier stages (Supplementary Fig. S5A–C,E–G). The pattern was rescued by the exogenous administration of ecdysone to *smt3i* larvae (Supplementary Fig. S5D,H). Similarly, Achaete (Ac) and Senseless (Sens) expression in *PGX* discs is similar to their expression in younger discs (Supplementary Fig. S5I–K,M–O); Likewise, ecdysone administration was able to restore later stage expression patterns (Supplementary Fig. S5L,P). Taken together, these results confirm that ecdysone signaling, necessary for the progression of patterning in the discs, is reduced in *smt3i* and *PGX* discs.

### Low levels of ecdysone affect cell growth and proliferation but not cell death

**Figure 3:**
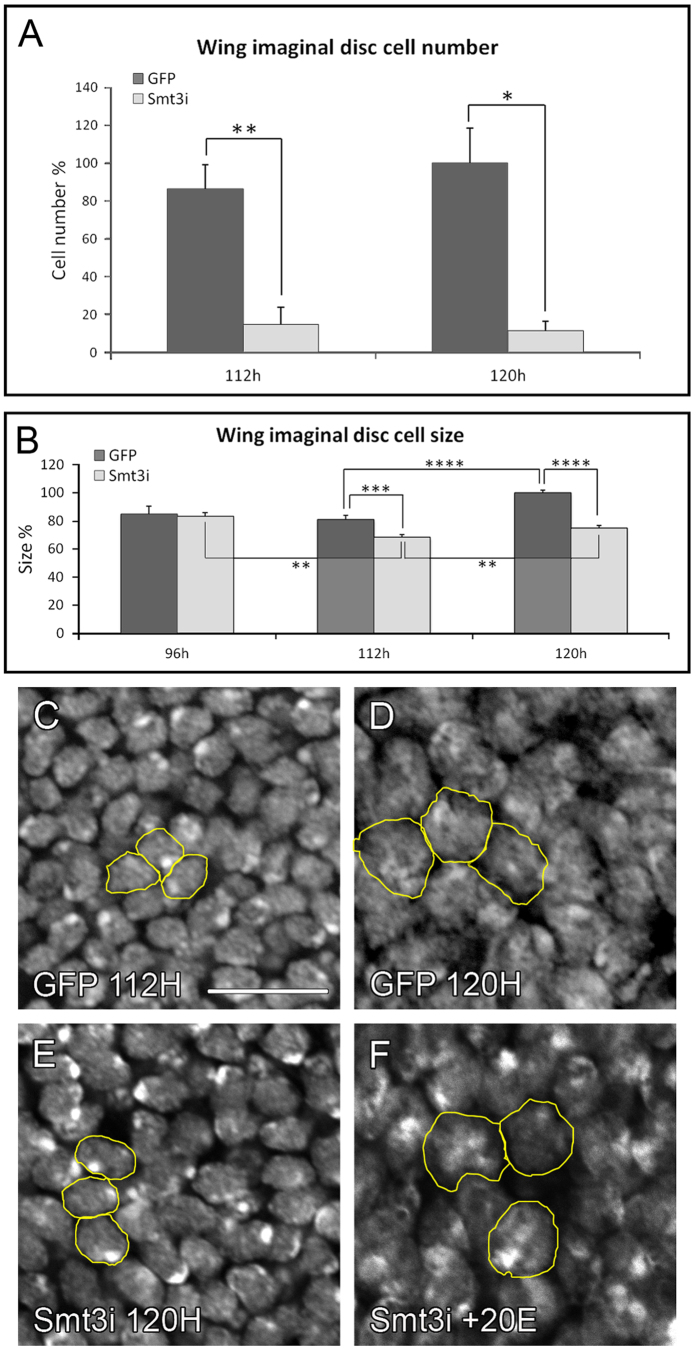
Cell number and cell size are reduced in wing discs exposed to low ecdysone levels. (**A**,**B**) Number of cells (**A**) and cell size (**B**) of wing discs from L3 *GFP* or *smt3i* larvae. Measurements are expressed in percentage relative to 120 hours AEL control discs. Error bars indicate standard deviation. One asterisk indicates p ≤ 0.05, two p ≤ 0.01, three p ≤ 0.001, and four p ≤ 0.0001. (**C**–**F**) Single plane confocal pictures of cell nuclei from wing discs, from *GFP* (**C**,**D**) or *smt3i* larvae (**E**,**F**), either with (**F**) or without (**C**,**E**) exogenous ecdysone administration (+20E). Scale bar indicates 10 μm. All pictures were taken under the same conditions. Some representative nuclei are outlined in yellow.

To understand whether the observed differences in size were due to defects in proliferation, cell growth or cell death we analyzed whether reduced levels of ecdysone affect these parameters. We found that control discs contain 5 times more cells than those from *smt3i* larvae (Fig. 3A). To confirm whether these results arise from reduced proliferation rate, we immunostained discs for phospho-Histone 3 (pH3) and found that the mitotic index was significantly reduced in discs of *smt3i* larvae when compared to controls (Supplementary Fig. S6A–C,E).

Our results also showed that control cells undergo a significant size increase from 112 to 120 hours AEL (Fig. 3B–D). However, cells from *smt3i* larvae decrease in size between 96 and 112 hours AEL, and this difference in size is maintained at 120 hours AEL (Fig. 3B,E). In addition, while most of the 120 hours AEL control cells are in G2 phase of the cell cycle, there is an increase in the number of cells arrested in G0/G1 phase in *smt3i* (data not shown), supporting the pH3 staining results.

We also analyzed whether low-ecdysone levels induce cell death by immunostaining with anti-activated-caspase-9 or -3 antibodies. We did not detect differences among the discs from controls, *smt3i* or *PGX* larvae (data not shown). Taken together, we conclude that the small size of *smt3i* wing discs is a consequence of a reduction in cell proliferation and size, but not of increased cell death. Therefore, cell proliferation and cell size appears to be promoted by ecdysone in mid-late L3.

**Figure 4:**
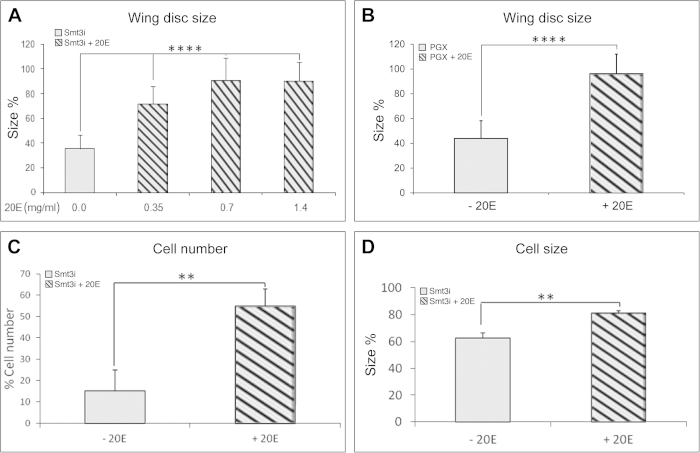
Wing disc size is rescued by exogenous ecdysone administration. (**A**,**B**) Relative area of 120 hours AEL *smt3i* (**A**) or 42 hours AL3E *PGX* (**B**) larvae wing discs before and after exogenous ecdysone administration (+20E). (**C**,**D**) Cell number (**C**) or cell size (**D**) of *smt3i* wing discs rescued with ecdysone. Error bars represent standard deviation. Measurements are expressed in percentage relative to 120 hours AEL *GFP* discs (**A**,**C**,**D**) or 42 hours AL3E* > Grim* discs (**B**). Two asterisks indicate p ≤ 0.01, four p ≤ 0.0001.

To verify that the growth phenotype of wing discs from *smt3i* larvae is due to the low levels of ecdysone, we conducted rescue experiments by adding ecdysone to the food and saw that the exogenous administration of ecdysone could induce growth (Fig. 4A). The recovery of wing size correlated with the amount of ecdysone (Fig. 4A). Similarly, ecdysone also restored *PGX* larvae wing disc size to that of control discs (Fig. 4B).

The exogenous ecdysone administration was able to restore the cell proliferation rates (Supplementary Fig. S6D,E). As a consequence, there is an increase in the cell number of discs from *smt3i* larvae (Fig. 4C). Furthermore, cell size is also increased (Figs 3F and 4D). Therefore, we can conclude that ecdysone promotes cell growth in addition to cell proliferation in the mid-late L3 discs.

### IIS/Tor signaling is altered in discs of larvae with low ecdysone levels

To explore the mechanism responsible for the lack of growth when ecdysone levels are reduced, we analyzed two pathways known to interact with ecdysone to regulate systemic growth, the *Dm*^4^ and the Juvenile Hormone (JH) signaling pathways^28^,^29^. Our results showed that Dm RNA or protein expression levels are moderately higher in *smt3i* discs compared to WT, but that this expression is reduced after exogenous ecdysone administration (Supplementary Fig. S7A,B). In addition, levels of *Krüppel-homolog 1* (*Kr-h1*), a key mediator of JH, did not show significant differences between control or *smt3i* larvae discs by qPCR (Supplementary Fig. S7C). These results do not support the hypothesis that reduced Dm or increased JH signaling accounts for slow growth of the *smt3i* discs.

The IIS pathway is able to control the growth of the organism by orchestrating cell growth and proliferation^3^. Therefore, we explored the production or release of the dIlps by immunostaining using anti-dIlp2 antibodies in L3 control and *smt3i* larvae. Our results showed that production and release of dIlp2 is not compromised in *smt3i* larvae (Supplementary Fig. S7E–H). Similar results were obtained in *PGX* larvae or by using anti-dIlp5 antibodies (data not shown).

Despite the fact that dIlp2 seemed to be produced and released at normal levels, alterations in ecdysone signaling could affect levels of IIS/Tor signaling in the discs. Phosphorylation of Akt1 (pAkt1) in response to insulin is often used as readout of the IIS pathway. We checked the levels of pAkt1 in discs by Western blot. In control discs, pAkt1 levels decreased after the ecdysone pulse (Supplementary Fig. S7D, GFP 120 *vs.* 112 hours AEL). In *smt3i* larvae of the same age, pAkt1 levels were significantly higher than in controls and decreased after exogenous ecdysone administration. Therefore, we conclude that the IIS pathway is activated at the level of pAkt1 in *smt3i* discs and that the changes seen in pAkt1 in mid L3 are unlikely to cause the reduced cell size and proliferation rates of *smt3i* larvae.

**Figure 5:**
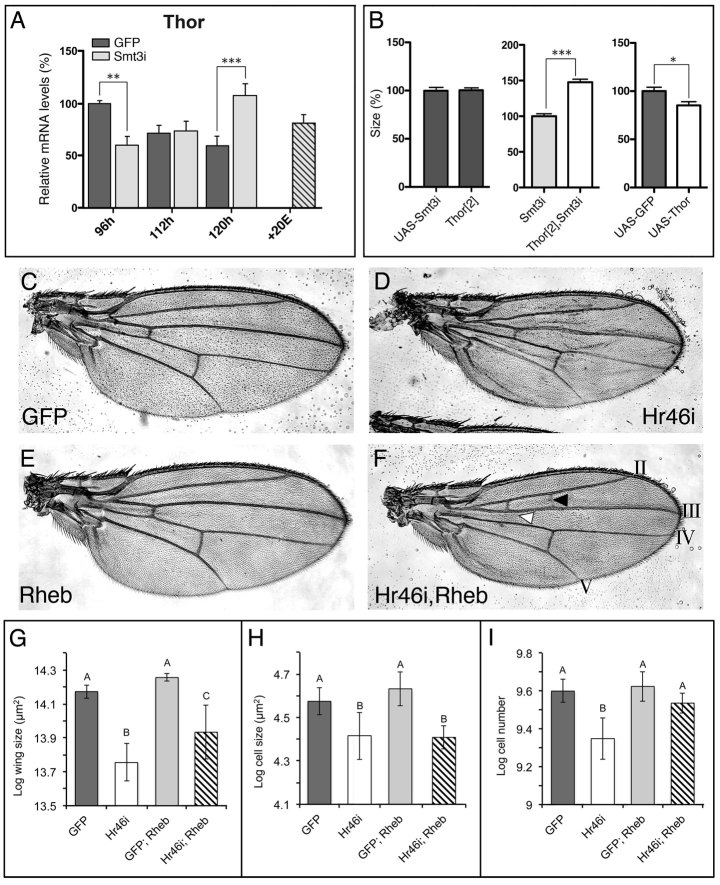
The effect of ecdysone on wing disc growth is mediated by the Tor pathway. (**A**) qPCR analysis of *Thor* transcription in wing imaginal discs. Error bars indicate standard deviation. Values are expressed in percentage relative to 96 hours AEL control discs. (**B**) Average size of wing imaginal discs at 120 hours AEL of the indicated genotypes. Over-expression of the indicated UAS transgenes was driven by *MS1096-Gal4* at 29 °C. One asterisk indicates p ≤ 0.05, two p ≤ 0.01 and three p ≤ 0.001. (**C**–**F**) Microphotographs of adult wings of the indicated genotypes showing the growth phenotype produced by the silencing or over-expression of the indicated genes driven by *MS1096-Gal4*. In F, black arrowhead indicates ectopic crossveins formed between longitudinal veins LII and LIII and white arrowhead indicates the absence of the anterior crossvein between veins LIII and LIV. (**G**–**I**) Graphical representation of the logarithm of the average adult wing size (**G**), cell number (**H**) and cell size (**I**) with expression of the indicated UAS-transgenes driven by *MS1096-Gal4.* There is a significant epistatic interaction between *Hr46* knock-down and Rheb overexpression on wing area (**G**) and cell number (**H**) (ANOVA, *Hr46i:*Rheb interaction, n = 154 and 39, *P* = 0.0238 and 0.0021, respectively). There is no significant interaction between *Hr46* knock-down and Rheb overexpression on cell size (**I**) (ANOVA, *Hr46i:*Rheb interaction, n = 39, *P* = 0.1987). Columns with different letters are significantly different (Tukey HSD, *P* < 0.05). Error bars represent standard deviation.

We wondered whether IIS/Tor pathways components downstream of pAkt could be altered in *smt3i* discs. A recent report showed that the genital discs of *Drosophila* have reduced sensitivity to nutrition due to repression of the transcription factor Forkhead box, sub-group O (Foxo), while components of IIS/Tor signaling upstream of Foxo remain unaltered^30^. In a similar way, although pAkt1 appears increased in the *smt3i* discs, components downstream of pAkt1 might show reduced activity in response to low ecdysone titers. The IIS/Tor pathways ultimately inactivate Thor, a negative growth regulator that inhibits translation through binding of the Eukaryotic Initiation factor 4F (eIF4F)^31^,^32^,^33^. In control discs, *Thor* expression levels were high at 96 hours but decrease at 112 and 120 hours AEL (Fig. 5A). This is in agreement with previous reports that saw low expression of *Thor* in late L3 discs^34^. However, discs derived from *smt3i* larvae showed significantly increased levels of *Thor* at 120 hours AEL (Fig. 5A). Furthermore, exogenous administration of ecdysone restored *Thor* mRNA to the control levels (Fig. 5A). These data suggests that ecdysone negatively regulates *Thor* expression and that reduced growth of discs in *smt3i* larvae might be due to increased Thor activity. To test whether Thor is necessary for growth suppression in *smt3i* discs we measured the effect of removal of ecdysone in a *Thor* mutant background. Consistent with our hypothesis, growth of discs in *smt3i* larvae was partially, but significantly rescued (Fig. 5B). Conversely, overexpression of *Thor* in the wing discs significantly reduced their size (Fig. 5B). These data suggest that ecdysone exert its positive role on growth though *Thor* regulation.

To further explore the connection between the ecdysone and the IIS/Tor pathways, we therefore conducted an epistatic analysis and manipulated each pathway, alone and in combination, specifically in the wing imaginal disc. To reduce ecdysone signaling in the wing imaginal disc, we used the *MS1096-Gal4* driver to knock-down expression of the ecdysone-downstream gene *Hr46.* To up-regulate the activity of IIS/Tor, we overexpressed the Ras homolog enriched in brain ortholog (Rheb), a negative regulator of Thor, using the same driver. Consistent with the hypothesis that ecdysone signaling promotes disc growth, knock-down of *Hr46* during development reduced adult wing size (Fig. 5D,G). However, the negative effect of *Hr46* on wing size was significantly reduced in wings where Rheb was also over-expressed (Fig. 5F,G). Importantly, our results show a significant epistatic interaction between the effect of *Hr46* knockdown and *Rheb* overexpression on adult wing size. Similar results were obtained with the overexpression of *Rheb*, an active form of the Ribosomal Protein S6-p70-protein kinase or other components of the IIS/Tor pathways using *apterous-Gal4* (data not shown).

To investigate whether the phenotypes obtained were due to a change in cell number or cell size, we counted the number of bristles in a constant area between the longitudinal veins LIV and LV of each wing. Each cell of the wing produces a single bristle; therefore counting the number of bristles in a known area of the wing allows us to calculate the area of each cell. Further, we can estimate the number of cells in a wing by dividing total wing area by cell area. We found that knockdown of *Hr46* reduced both wing cell size and wing cell number (Fig. 5H–I). Intriguingly, overexpression of Rheb rescued the effect of *Hr46* knockdown on cell number (Fig. 5H), but did not rescue the effect of *Hr46* knockdown on cell size (Fig. 5I). Collectively these data support the hypothesis that ecdysone exerts organ-autonomous effects on imaginal disc growth via *Thor-*mediated regulation of cell number. In addition, ecdysone affects disc growth via an unknown regulator of cell size.

### Role of ecdysone in organ growth is evolutionarily conserved

**Figure 6:**
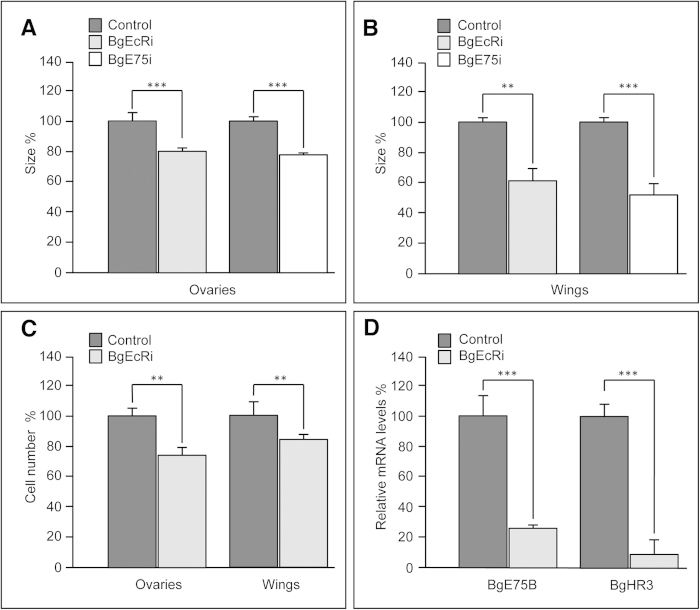
Ecdysone promotes organ growth in hemimetabolous insects. (**A**,**B**) Area of ovaries and wings from control, *BgEcR* or *BgE75* dsRNA-treated nymphs at the end of the last nymphal stage. (**C**) Cell number in 8-days-old ovaries and 5-days-old wings from control and *EcRi* last instar nymphs. (**D**) qPCR analysis of the indicated genes using mRNA from control or *EcRi* wings from 5-days-old last instar nymphs. Error bars indicate standard deviation. Measurements are expressed in percentage relative to control organs. Two asterisks indicate p ≤ 0.005, three p ≤ 0.001.

To address whether ecdysone also controls organ growth in more primitive insects, we turned to the cockroach *Blattella germanica* as a model of hemimetabolous development. In this species, the metamorphic transition, which occurs in the last (N6) nymphal instar, is characterized by a significant growth of wings and ovaries^35^,^36^. To examine the role of ecdysone in the growth of these tissues, *BgEcR* dsRNA was injected into freshly ecdysed last instar nymphs to reduce the levels of the ecdysone receptor. Wings and ovaries from *BgEcRi* nymphs showed a significant reduction in growth when compared to nymphs treated with control dsRNA (Fig. 6A,B). We also analyzed tissue growth in last instar nymphs with reduced ecdysone levels obtained by silencing the *BgE75* nuclear receptor, which causes premature degeneration of the PG^37^. Consequently, growth of wings and ovaries was significantly impaired (Fig. 6A,B), mainly due to a significant decrease in cell number (Fig. 6C). Furthermore, the expression of the ecdysone-inducible genes *BgE75B* and *BgHR3* (homologous to *Hr46*) in the wings of *BgEcRi* nymphs was severely reduced (Fig. 6D). Taken together, these experiments reveal that ecdysone is required to promote organ growth in *B. germanica*, highlighting the conserved function of ecdysone in the control of organ growth in both hemimetabolous and holometabolous insects.

## Discussion

Steroid hormones regulate a wide variety of biological responses by activating target genes in a stage- and tissue-specific manner. In insects, ecdysone is the central regulator of developmental transitions. In *Drosophila*, pulses of ecdysone trigger each of the larval molts, the puparium formation and metamorphosis^38^. These ecdysone pulses are reminiscent of those of the mammalian sex steroid hormones that enable the transition from childhood to puberty to adulthood in humans^39^.

At the end of L3, dramatic changes occur due to ecdysone signaling, an example being the histolysis of most of the larval tissues. Conversely, ecdysone induces growth arrest in tissues that will form the adult organs and the processes of morphogenesis and differentiation. In contrast, we report here that increasing levels of ecdysone promote growth of imaginal tissues between 96 and 112 hours AEL. Interestingly, in low ecdysone levels at mid-late L3, *smt3i* imaginal discs reach their final size between 96 and 112 hours AEL, and do not grow significantly afterwards despite the dramatically extended larval period (Fig. 1H). These results allow us to speculate about the existence of a checkpoint for imaginal disc size that could coincide with a minor peak of ecdysone between 96 and 112 hours AEL or with the rise of the final peak of ecdysone before pupariation^16^,^38^, which could not be overcome in low ecdysone conditions. This checkpoint would ensure that organs restrain their growth under abnormal hormonal conditions.

The growth promoting effect of the mid-late L3 pulse of ecdysone described here is different from the previously reported roles of ecdysone on the control of tissue growth that take place during larval development. For example, ecdysone was shown to be a negative regulator of systemic growth in *Drosophila* through the inhibition of Dm in the FB^4^. In contrast, several studies show that ecdysone positively regulates organ growth in other species or in other developmental times. Mitchell and coworkers^40^ reported that cell cycle progression in the zone of non-proliferating cells of the wing disc of late L3 larvae in *Drosophila* was regulated by the ecdysone-inducible transcription factor Crooked legs. Furthermore, in *Drosophila* larvae where ecdysone synthesis is inhibited by damage to the wing discs, slow growth of the other discs is rescued by application of exogenous ecdysone^41^. Recently, it has been shown that varying the ecdysone levels by dosage manipulation of the microRNA *bantam* (*ban*) specifically in the PG produces different outputs^42^. A mild inhibition of ecdysone levels by overexpression of *ban* using *P0206-Gal4* rescues the wing imaginal disc size of *ban*^Δ*1*^ mutants, while a stronger inhibition using *phm-Gal4* phenocopies *smt3i* larvae. These differences in the role of ecdysone are also observed in Lepidoptera, in which moderate levels of ecdysone are required for epidermal and imaginal disc cell growth^43^,^44^,^45^,^46^, while higher concentrations stop proliferation^43^,^44^. All these results indicate that the fluctuations in the levels of the hormone are important to determine the nature of the response to ecdysone.

As mentioned, the role of ecdysone in organ growth is conserved in holometabolous insects^43^,^44^,^45^,^46^. Unlike holometabolous insects, in hemimetabolous insects, such as *B. germanica*, growth and maturation occur simultaneously throughout successive nymphal stages until the imaginal molt. Despite the differences in the developmental strategies between holometabolous and hemimetabolous insects, we have shown that ecdysone also controls growth in wings and ovaries of *B. germanica* nymphs demonstrating the conserved role of ecdysone in organ growth in winged insects.

The IIS/Tor pathways are nutrient-sensing pathways implicated in the control of growth. Each branch can act independently or together. Downstream of the dInR activation by dIlps, pAkt1 activates the Tor pathway through the inhibition of the tuberous sclerosis complex 2 (TSC2)^47^. The Tor pathway promotes translation through activation of S6k and inactivation of Thor^48^. The inhibition of Thor will promote the liberation of eIF4F, promoting the formation of the initiation complex and increasing global translation initiation^49^,^50^. Mutations in the components of this pathway lead to changes in cell size and number.

Our results suggest that ecdysone regulates the levels of Thor, which may affect IIS/Tor signaling in the discs downstream of the activation of pAkt1. Several lines of evidence support this conclusion: i) dIlps are being produced in the IPCs and secreted to the hemolymph in a similar way in *smt3i, PGX* and control larvae; ii) the *smt3i* discs do not show a reduction in the levels of pAkt1 respect to control larvae; iii) *smt3i* discs have increased levels of *Thor*; iv) mutation in *Thor* partially restores the size of smt3i discs, and v) the effects of wing-specific knock-down of *Hr46* on total wing size and wing cell number (but not wing cell size) are reduced or eliminated when accompanied by elevated expression of *Rheb*, a suppressor of *Thor*. Overall, decreasing ecdysone appears to affect the sensitivity of the discs to the dIlps by regulating the expression of downstream components. This role of ecdysone in promoting organ growth is in contrast to, but compatible with the role of ecdysone in controlling systemic growth by repressing Dm function in fat cells^4^.

Interestingly, ecdysone does not affect the activity of all components of the IIS/Tor pathways but appears to regulate a particular node to control imaginal tissue growth. Despite increased phosphorylation of Akt1, the IIS/Tor pathways seem to be downregulated in *smt3i* discs, as the levels of *Thor* expression are higher. In *Drosophila* genital discs, overexpressing IIS/Tor components upstream of Foxo have no effect on genital disc size, whereas manipulating Foxo expression increases the nutrition sensitivity of this disc^30^. This suggests that what happens upstream of Foxo is not important, as Foxo is the limiting factor in the genital imaginal discs. In a similar way, ecdysone could tune the sensitivity of the disc to IIS/Tor by increasing the levels of Thor. Upon phosphorylation, Thor dissociates from eIF4F permitting translation initiation and growth. Our results suggest that ecdysone has a role in the inhibition of *Thor* transcription in mid-late L3 larvae, promoting cell growth and proliferation in that stage. Since only a partial rescue of the imaginal discs size in a *Thor*^2^ mutant background was observed, this might reflect the participation of other unidentified factors regulated by ecdysone in the attainment of final size. Consistent with this hypothesis is the observation that suppression of Thor in wings with reduced ecdysone-signaling rescues cell number but not cell size.

The relationship between Thor and disc growth has been reported in the literature. Miron and coworkers^32^ have previously shown that the ectopic overexpression in wing discs of a mutant Thor that binds with high affinity to eIF4F produces a reduction of wing size caused by smaller cells and slower proliferation. We obtained a similar effect by overexpressing the WT form of Thor at 29 °C using a strong wing specific Gal4 driver. Mason-Suares and coworkers^51^ showed that Thor expression is regulated by Polycomb group factors. Polycomb repressive complex 2 (PRC2) subunit mutants show small discs, wing discs mutant for *Enhancer of zeste* [*E(z)*, a subunit of PRC2], being only 25% the size of WT wing discs^34^. However, in *E(z)* mutants, *Thor* expression is derepressed. Simultaneous removal of *Thor* and *E(z)* partially rescues the size of the discs. So Thor must be responsible, in part, for cell proliferation defects due to its role in suppressing cap-dependent translation and cell growth. Besides the wing disc, Thor may also be implicated in growth regulation during development of other imaginal discs.

Our results are also in agreement with the relationship between S6k and Hr46 reported by Montagne *et al.*^52^. We show that overexpression of components of the IIS/Tor pathways counteracts the reduction of growth phenotypes produced by knock-down of *Hr46*. These results indicate a requirement for both IIS/Tor and ecdysone to reach the final disc size and suggest the two pathways converge and share common players. This crosstalk would occur downstream of Akt1, with Thor being a plausible key element.

In summary, we report here the positive role of ecdysone in promoting organ growth in mid-late L3 larvae, which contrasts with the systemic negative role for the hormone in promoting cell differentiation and growth arrest. Ecdysone appears to converge with the IIS/Tor pathways in the regulation of Thor. Interestingly, the crosstalk between these pathways might contribute to the maintenance of the relative proportions among the different organs of an individual, as tissues need to compute inputs coming from different sources in order to proceed with growth during development.

## Methods

### *Drosophila melanogaster* strains

Flies were raised on standard *Drosophila* medium with or without bromophenol blue at 25 °C at low density. Strains: *phantom-Gal4,UAS-mCD8::GFP/TM6B,Tb* (*phm-Gal4*, P. Leopold)^18^,^19^, expressed in the PG; *w;MS1096-Gal4*^53^, expressed in wing and haltere imaginal discs; *C765-Gal4* (Bloomington- BDSC- B#36523), expressed in wing and leg imaginal discs; *UAS-smt3i*^20^; *UAS-Hr46i* (Vienna- VDRC- #v12044 and #v20157); *UAS-Rheb* (B#22248); *UAS-Dicer* (B#24650, B#2465); *w;UAS-grim*^54^; *w;UAS-Thor.wt* (B#9147); *w;UAS-EcR B1*^*w650*^ 55; *tub-Gal80*^*ts*^ (B#7017); WT (Vallecas); *w*^*1118*^ (B#5905); *y*^*1*^*w;Thor*^2^ (B#9559). Other strains can be found in FlyBase (http://flybase.bio.indiana.edu).

### *Drosophila melanogaster* larvae and fly collection

Eggs were collected for 4 hours at 25 °C on food containing bromophenol blue at 0.05% (w/v). Larvae were collected as described^56^: mid L3 96 hours AEL, early-wandering L3 112 hours AEL and late-wandering L3 120 hours AEL, with cleared guts and swollen salivary glands^57^. This time correspond to the highest levels of ecdysone before entering into pupariation in WT larvae. To analyze wings, flies were grown at 18 °C.

The procedure to genetically ablate the PG is detailed in Supplementary Fig. S1. Controls were *w*^*1118*^ females crossed with parental *w;UAS-Grim* (*> Grim*) or *w;phm-Gal4,tub-Gal80*^*ts*^ (*PG >*). PG ablation was induced by shifting larvae from 17 °C to 29 °C (the progeny at 29 °C is referred as *PGX*). As an additional control, we allowed larvae to develop continuously at 17 °C.

For exogenous ecdysone rescue experiments, *smt3i* blue gut larvae were collected at 112–120 hours AEL and placed in groups of 10 individuals on food supplemented with 20E (Sigma) dissolved in ethanol at 0.35, 0.7 or 1.4 mg/ml and mixed with yeast. For *PGX* larvae, 10 larvae were transferred at 0 h AL3E to standard food containing either 0.15 mg of 20E (dissolved in ethanol)/ml of food, or an equivalent volume of ethanol (control), and left to feed for 42 hours.

### RNAi interference in *B. germanica*

*B. germanica* specimens were reared in the dark at 30 °C and 60–70% relative humidity. RNAi *in vivo* in nymphs of *B. germanica* was performed as described^58^,^59^. dsRNAs against *BgEcR* or *BgE75* targeted a 529-bp or a 612-bp fragment, respectively. Control dsRNA was a non-coding sequence from the pSTBlue-1 vector. Primers are indicated in Supplementary Table S1. 1 μl of dsRNA solution (4 μg/μl) was injected into the abdomen of newly emerged last instar nymphs, previously anesthetized with carbon dioxide.

### Immunocytochemistry, organ size measurement and mitotic index calculation

96, 112 or 120 hours AEL larvae were collected and stained as described^21^. Antibodies: rabbit anti-Dm (1:5000)^60^; rabbit anti-pH3 (1:50; Santa Cruz, SC-8656-R); mouse anti-Wg (1:5; Developmental Studies Hybridoma Bank- DSHB- #4D4); mouse anti-Ct (1:5; DSHB #2B10); mouse anti-Ac (DSHB); guinea pig anti-Sens (H. Bellen, Houston, USA); rat anti-Ilp2 (1:800)^8^; rat anti-Ilp5 (1:800)^8^; goat anti-Caspase 9 (1:10; Santa Cruz, SC-2218); rabbit anti-Caspase 3 (Cell Signaling #9661); fluorescent Alexa 568 and 488 (1:200; Molecular Probes). DAPI (Roche) was used at 1:2000. Confocal images were taken with a Leica DM-IRE2 or Zeiss LSM-510 microscope. Organ sizes were calculated using ImageJ. Statistical analysis was done using GraphPad PRISM.

Adult wings were dissected in EtOH and mounted in Lactic Acid:EtOH, 6:5. Pictures were taken under the same objective (4X) and size was calculated using ImageJ. Cell size was estimated by counting the number of bristles in a 105 μm × 105 μm square between the longitudinal veins LIV and LV of each wing. Wing cell number was estimated by dividing wing area by cell area.

For calculation of the mitotic index, the percentage of pH3 positive cells *versus* total number of cells (visualized with DAPI) was calculated for the same area in confocal pictures taken with 63x objective.

Dissections of *B. germanica* nymphal wings and ovaries were carried out in Ringer’s saline on carbon dioxide-anesthetized specimens. Tissues were fixed in 4% paraformaldehyde, permeabilized in PBS-0.2% Tween 20 (PBTw), incubated for 10 minutes in 1 μg/ml DAPI in PBT and washed twice with PBTw. Proliferation was monitored by *in vivo* labeling with 5′-bromo-2-deoxyuridine (BrdU) as described^58^. Images were taken with an AxioImager.Z1 (Zeiss) microscope and processed using Adobe Photoshop.

### Wing disc cell size and cell number analysis

Wing discs were isolated in PBS from 20–40 specimens per genotype. Discs were disaggregated in 500 μl of Trypsin-EDTA (Sigma), stirring for 1 hour, adding 1 μl of Draq5 (Abcam), stirring for another hour and adding 10 μl of fetal bovine serum. The solution was filtrated through a 30 μm nylon filter and applied to a Becton Dickinson FACS Canto II for cell size calculation. For cell counting, 10 μl of a 1:1 mix of a filtered solution:trypan blue stain at 0.4% was placed in a Countess automated cell counter (Invitrogen). At least three different samples were measured per genotype.

### Statistical Analysis

Statistical analysis was conducted using GraphPad PRISM and JMP. Epistasis was detected by two-way ANOVA on the basis of the interaction between the effects of two genetic manipulations on phenotype (wing area, wing cell area and wing cell number). Wing area, cell area, and cell number data were log transformed prior the ANOVA.

### qPCR analysis

*Drosophila* RNA was extracted from isolated 4-8 wing discs per genotype. Total RNA was obtained using the “mirVana miRNA isolation kit” (Ambion). cDNA preparation and qPCR was performed as described^21^. Reactions were run in triplicate, using *RpL32* as control, and checked by electrophoresis. *Blattella* total RNA was isolated with the GenElute^TM^ Mammalian Total RNA kit (Sigma). cDNA preparation and qPCR analysis were conducted as described^36^. Primer sequences are indicated in Supplementary Table S1.

### Western blot analysis

Approximately 25 wing discs were collected in 20 μl of lysis buffer [300 mM NaCL, 50 mM Tris-HCl pH 7.5, 1% triton X-100, 1x phosphatase inhibitor (PhosSTOP, Roche), 1x protease inhibitor (Sigma)], centrifuged at 13000 rpm for 15 minutes at 4 °C, and the supernatant was recovered. Antibodies: rabbit anti-phospho-Akt1 (ser505; Cell Signaling, #4054); anti-Dm P4C4 B10 (1:50)^61^; anti-β-tubulin E7 (1:1000; DSHB); HRP-conjugated secondary anti-rabbit and anti-mouse (1:2000–5000; Jackson ImmunoResearch). Three blots were quantified per antibody using ImageJ.

## Additional Information

**How to cite this article**: Herboso, L. *et al.* Ecdysone promotes growth of imaginal discs through the regulation of Thor in *D. melanogaster.* *Sci. Rep.* **5**, 12383; doi: 10.1038/srep12383 (2015).

## Supplementary Material

Supplementary Figure Legends

Supplementary Figure S1

Supplementary Figure S2

Supplementary Figure S3

Supplementary Figure S4

Supplementary Figure S5

Supplementary Figure S6

Supplementary Figure S7

Supplementary Table S8
