# Supplementary material for: Ecdysone promotes growth of imaginal discs through the regulation of Thor in D. melanogaster
Source: Sci Rep. 2015 Jul 22;5:12383. doi: 10.1038/srep12383 (PMC4510524; doi:10.1038/srep12383)
Supplement: Supplementary Figure Legends [file srep12383-s1.doc]

**Supplementary Information**

**Ecdysone promotes growth of imaginal discs through the regulation of Thor in *D. melanogaster***

Leire Herboso, Marisa M. Oliveira, Ana Talamillo, Coralia Pérez, Monika González, David Martín, James D. Sutherland, Alexander W. Shingleton, Christen K. Mirth and Rosa Barrio^*^.

**Supplementary Figure Legends**

**Supplementary Figure S1 The gut does not depend on ecdysone.** Bright field pictures of dissected guts from control (*UAS-smt3i*) or *smt3i* larvae taken under the same conditions (objective 4x; Several pictures were taken per gut). Anterior is to the left. Days (d) AEL are indicated. gc: gastric caeca; Mt: Malpighian tubules.

**Supplementary Figure S2 Method used to ablate the prothoracic gland at the beginning of the third instar.** (A) Genotypes used to ablate the PG. We used a PG-specific Gal4 to drive expression of a cell death-regulatory gene (*Grim*). To prevent cell death early in development and to restrict the study to where most imaginal tissue growth occurs, the third instar, we controlled the tissue ablation using *tub-Gal80^ts^*. (B) *tub-Gal80^ts^* is ubiquitously expressed by the *tub* promoter and represses Gal4 at 17°C allowing for normal development. At 29°C *tub-Gal80^ts^* is inactive and, consequently, through temperature shifts we induce ablation of the PG at specific times. (C) We reared all animals at 17°C from egg until the beginning of the L3, collected newly molted animals at two-hour intervals and shifted them to 29°C for tissue ablation. (D) Time scale equivalence between *PGX* and *smt3i* larval developmental times. All parts of this figure were drawn by the authors MMO and CKM.

**Supplementary Figure S3 The *PGX* larvae exhibit a reduced and deformed prothoracic gland and continue to grow.** (A-G) PGs (orange arrows) from *>Grim* (A, D, E), *PG>* (B) and *PGX* (C, F, G) larvae shifted to 29°C at the molt to the L3 and collected at 42 hours AL3E for tissue dissection and immunocytochemistry with Ac or Wg antibodies. Scale bar (G) indicates 100 µm. (H) *PGX* larvae grow at faster rates than controls. *>Grim, PG>* and *PGX* female larval weight at 0, 24 and 42 hours AL3E and *PGX* at 96 and 120 hours AL3E (4 and 5 days as third instar) is indicated. Measures are characterized by mean and 95% confidence intervals. Letters represent significant differences between genotypes (p≤0.01, Wilcoxon rank test using Holm’s p-value adjustment).

**Supplementary Figure S4 Ecdysone Receptor is necessary for wing imaginal disc growth.** Graphical representation of the size of wing imaginal discs at the indicated hours AL3E, overexpressing either a control transgene (GFP) or a dominant negative form of the ecdysone receptor (EcR DN) via the *C765-Gal4* driver. Error bars indicate standard error of the mean. One-way analysis of the variance ANOVA and Bonferroni's Multiple Comparison Test were applied. Three asterisks indicate p≤0.001.

**Supplementary Figure S5 Wingless, Cut, Senseless and Achaete expression in the wing discs depends on the ecdysone levels.** (A-P) Single plane confocal pictures of wing discs stained for Wg (A-D), Ct (E-H), Sens (i-l) or Ac (M-P) taken under the same settings per antibody. (A, B, E, F) The expression of Wg and Ct increases after the peak of ecdysone (120 hours AEL) in the control conditions (GFP). (C, G) In the case of *smt3i* larvae, the expression of Wg and Ct at 120 hours AEL is similar to the pattern sawn in younger disc. (I-K, M-O) *PGX* wing discs show delayed Sens and Ac expression pattern (K, O) compared to the controls (I, J, M, N). (D, H, L, P) Exogenous ecdysone administration (+20E) recovers the expression pattern of Wg, Ct, Sens and Ac in late L3 wing discs. Scale bar in P indicates 100 µm in I to P.

**Supplementary Figure S6 Proliferation is reduced in imaginal discs exposed to low ecdysone levels.** (A-D) Single plane confocal pictures of wing discs stained for anti-pH3, taken under the same conditions. (E) Graphical representation of the mitotic index. Control wing discs cells (GFP) proliferate significantly more than *smt3i* cells, but exogenous ecdysone (20E) administration increases their proliferation. Error bars indicate standard deviation. p-value was calculated by Student’s t-test. Two asterisks indicate p≤0.01, three p≤0.001.

**Supplementary Figure S7 Insulin liberation is not affected in low-ecdysone larvae.** (A, C) Q-PCR analysis of *dm* (A) or *Kr-h1* (C) expression in wing discs from *GFP* or *smt3i* larvae at 96, 112 or 120 hours AEL. Error bars indicate standard deviation. Values are expressed in percentage relative to 96 hours AEL control discs. (B, D) Western blot showing Dm (B) and pAkt1 (D) expression in wing discs from *GFP* larvae at 112 hours or 120 hours AEL and *smt3i* larvae fed with normal food or food supplemented with exogenous ecdysone (+20E). β-tubulin was used as loading control. Molecular weight markers are shown to the left. The relative intensity of the bands from three experiments in relation to β-tubulin is indicated in the graphic. 120 hours AEL *GFP* larvae are considered as 100%. Error bars indicate standard error of the mean. One-way analysis of the variance ANOVA and Bonferroni's Multiple Comparison Test were performed. One asterisk indicates p≤0.05, two p≤0.01, and three p≤0.001. (E-H) Single plane confocal pictures of L3 larval brain taken under the same conditions. Expression of dIlp2 is shown in the IPCs (surrounded with a yellow line), either under fed (E, G) or starvation conditions (F, H).
