# Supplementary figures and images for: Ecdysone promotes growth of imaginal discs through the regulation of Thor in D. melanogaster
Source: Sci Rep. 2015 Jul 22;5:12383. doi: 10.1038/srep12383 (PMC4510524; doi:10.1038/srep12383)

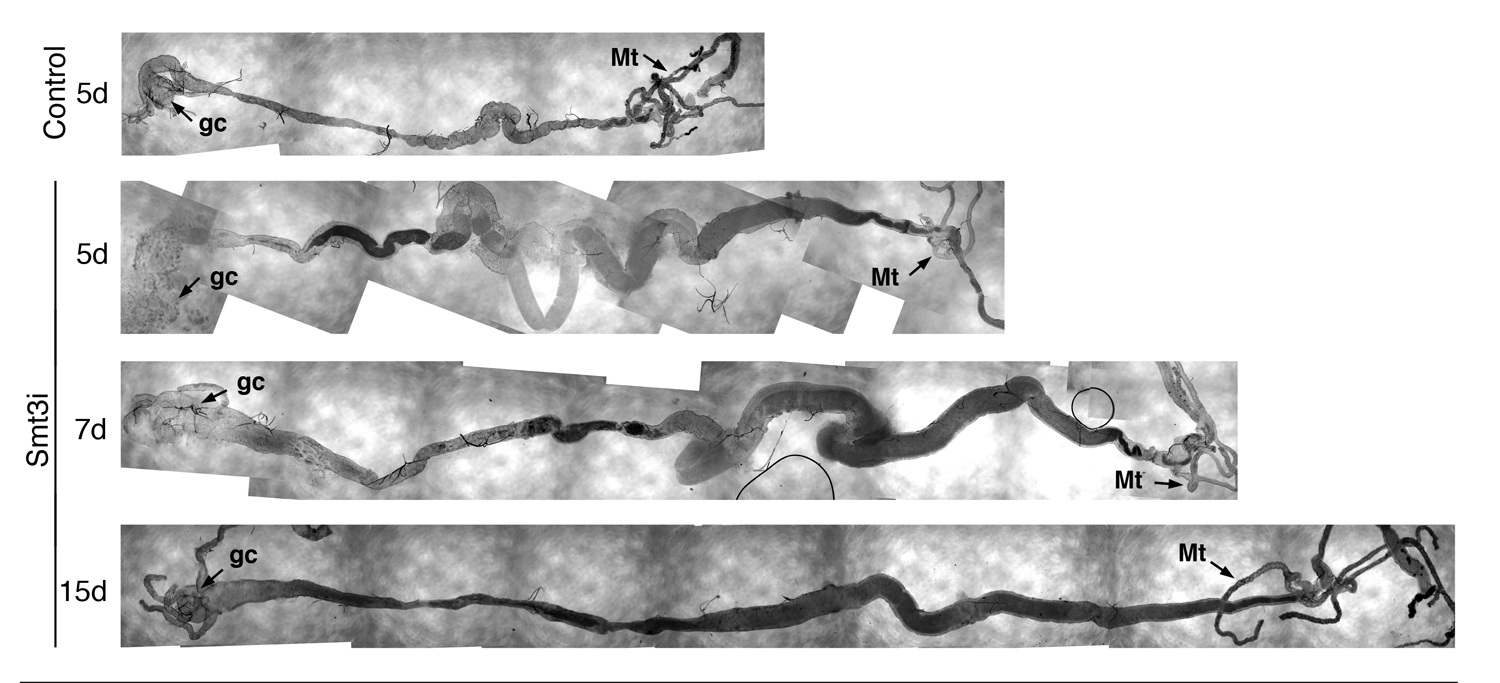

Supplement: Supplementary Figure S1 [file srep12383-s2.jpg]

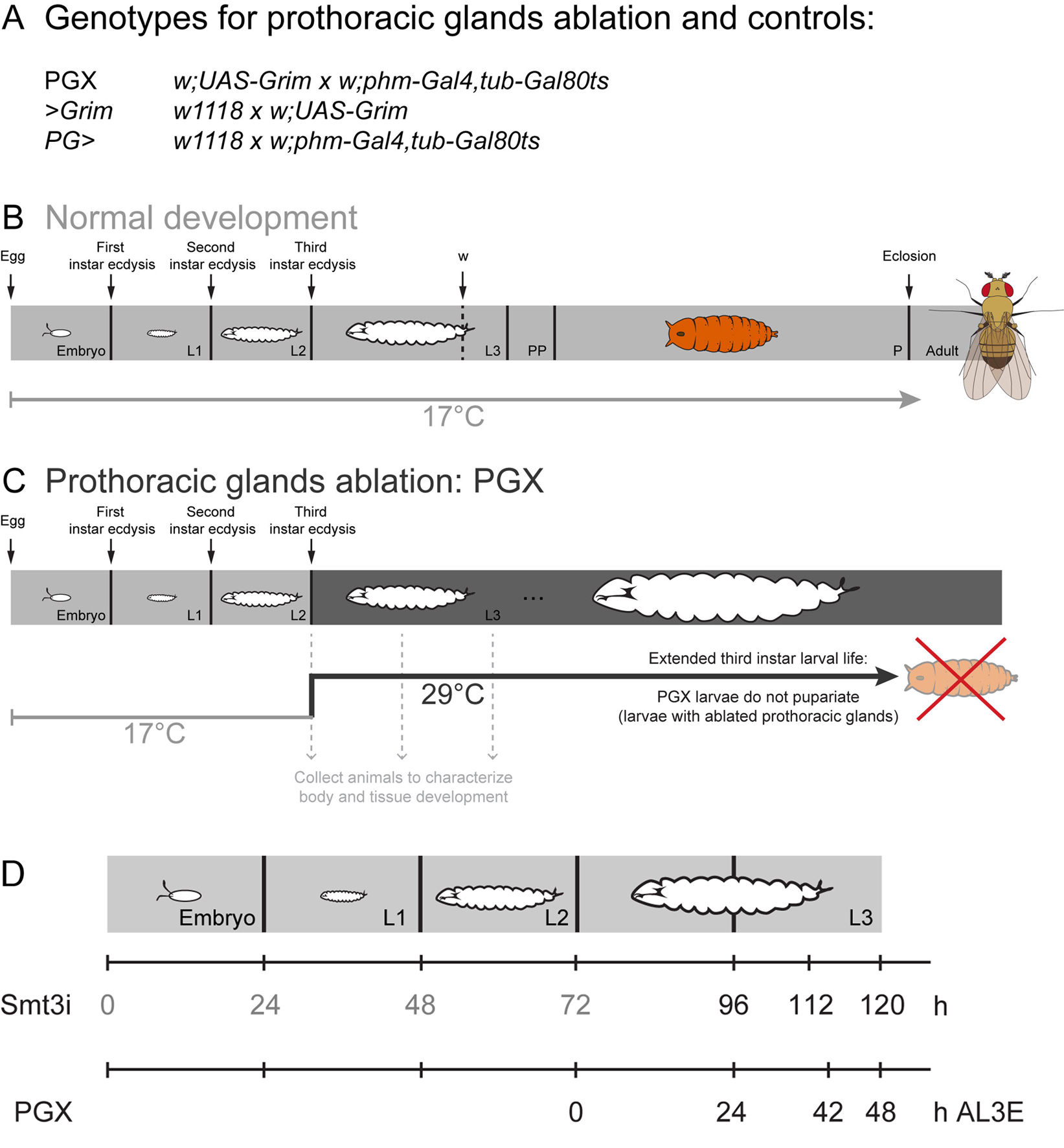

Supplement: Supplementary Figure S2 [file srep12383-s3.jpg]

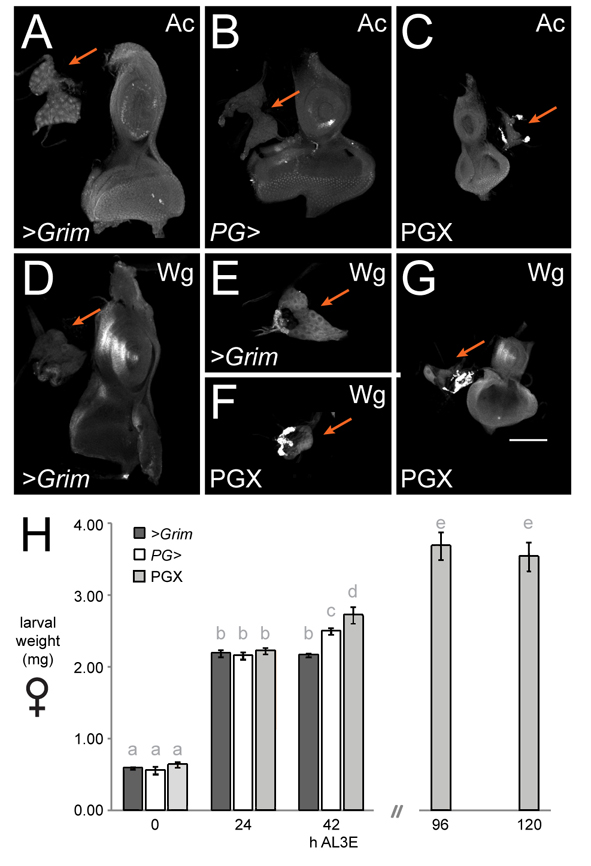

Supplement: Supplementary Figure S3 [file srep12383-s4.jpg]

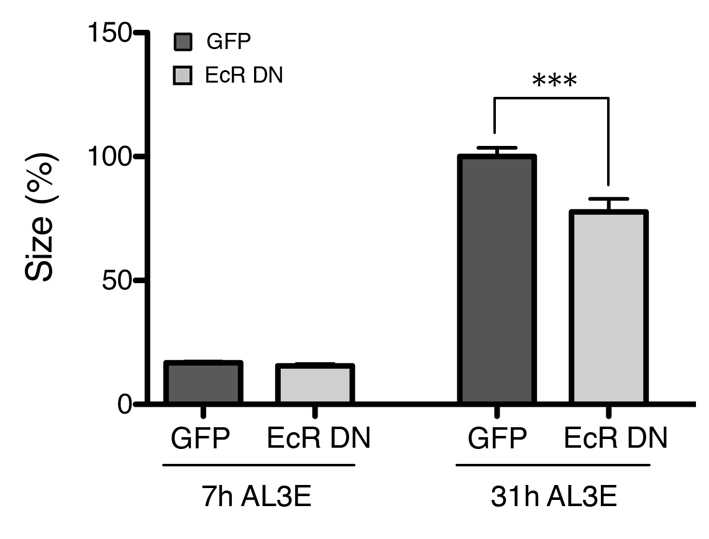

Supplement: Supplementary Figure S4 [file srep12383-s5.jpg]

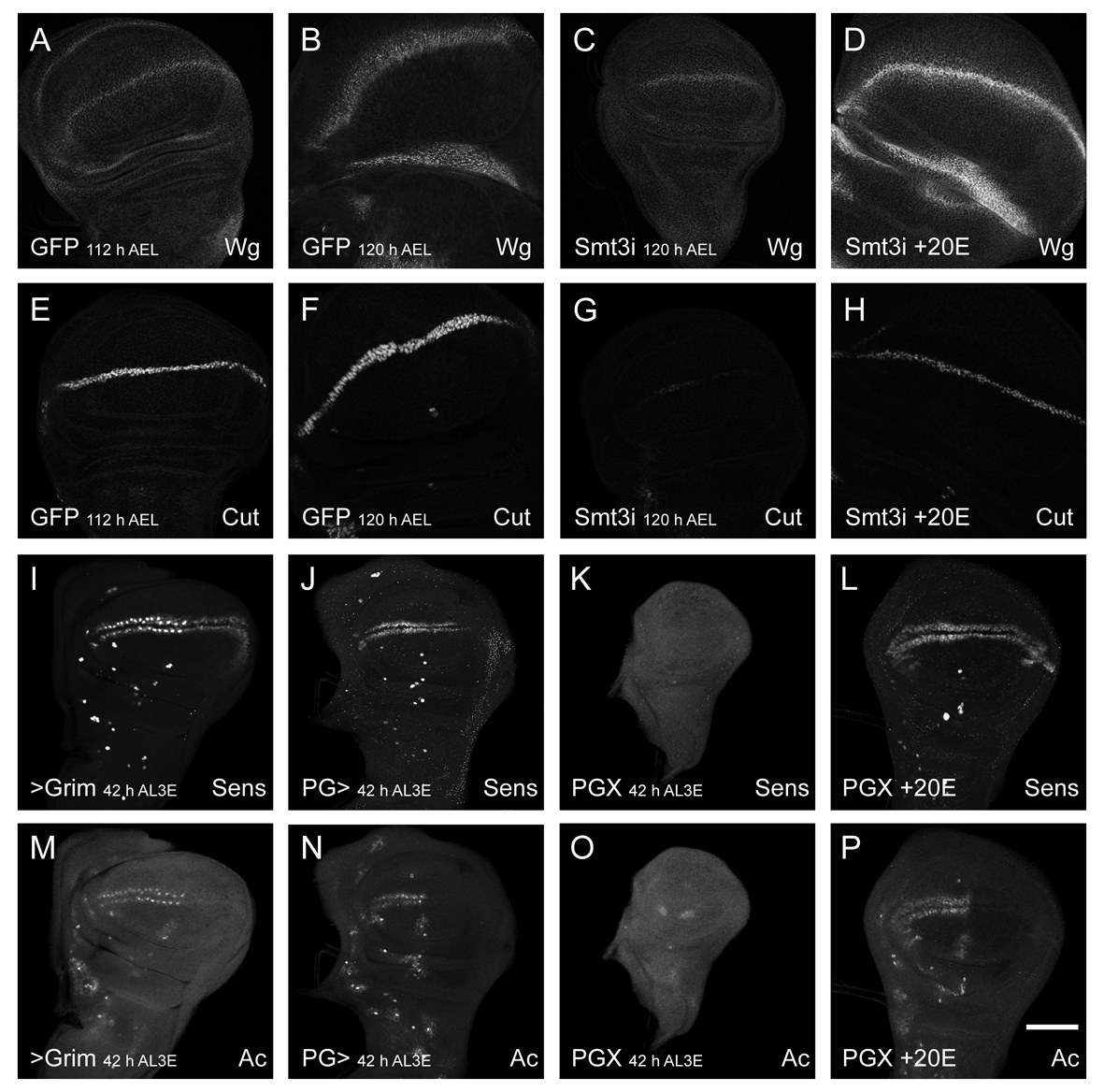

Supplement: Supplementary Figure S5 [file srep12383-s6.tiff]

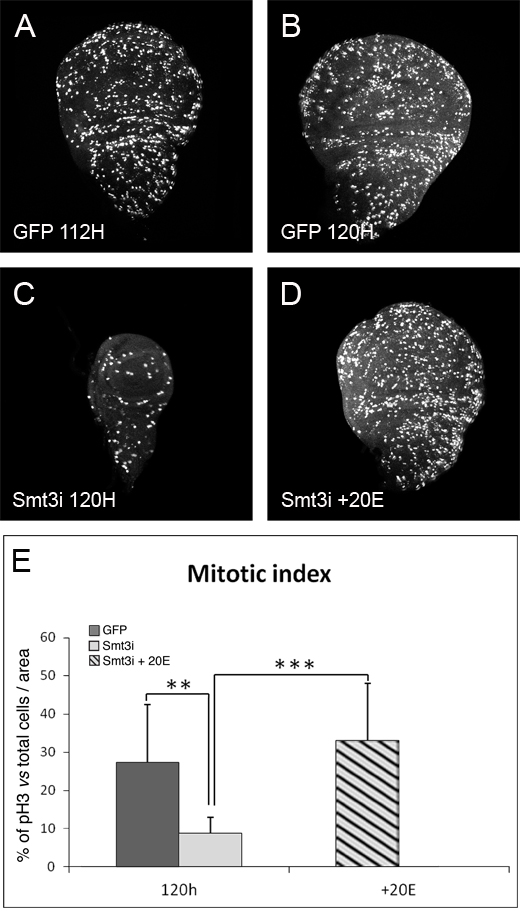

Supplement: Supplementary Figure S6 [file srep12383-s7.jpg]

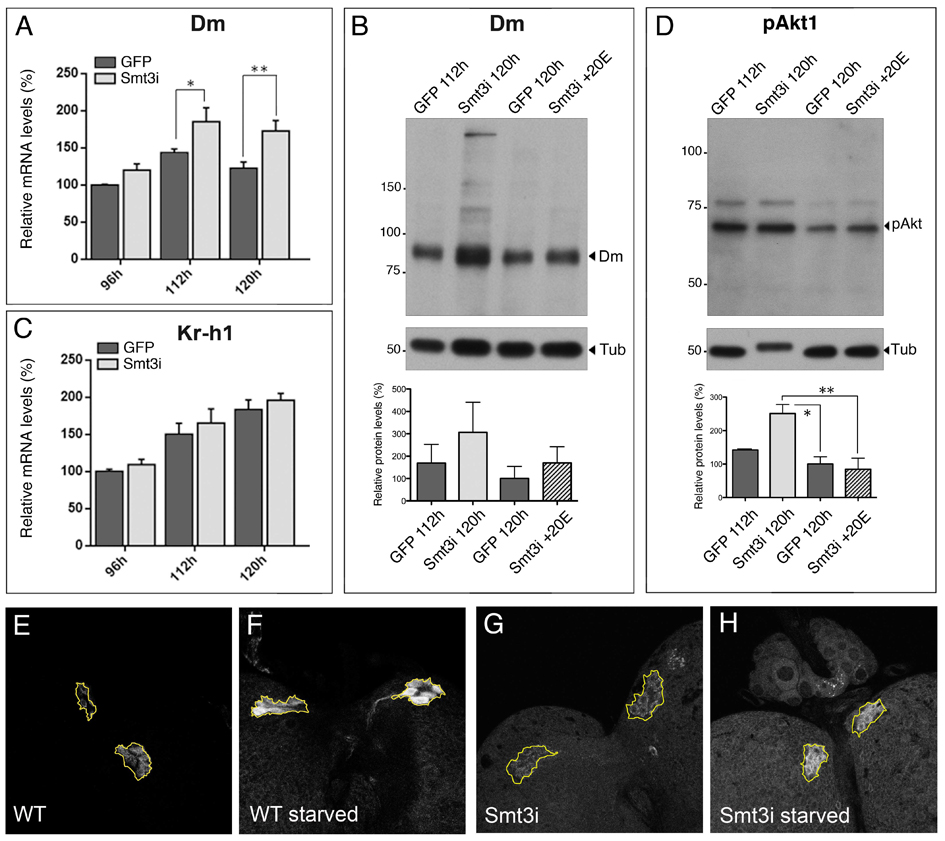

Supplement: Supplementary Figure S7 [file srep12383-s8.jpg]
