# Supplementary material for: Ecdysone promotes growth of imaginal discs through the regulation of Thor in D. melanogaster
Source: Sci Rep. 2015 Jul 22;5:12383. doi: 10.1038/srep12383 (PMC4510524; doi:10.1038/srep12383)
Supplement: Supplementary Table S8 [file srep12383-s9.docx]

**Supplementary Table S1. Oligonucleotide sequences.**

| **Name** ^1, 2^ | **Sequence** | **Reference** |
| --- | --- | --- |
| BgActin5C-Fwd | 5’-AGCTTCCTGATGGTCAGGTGA-3’ | This work |
| BgActin5C-Rev | 5’-TGTCGGCAATTCCAGGGTACATGGT-3’. | This work |
| BgE75B-Fwd | 5’-CTAACGCTCCGCAATCCAGTTCA-3’ | This work |
| BgE75B-Rev | 5’-GTGAATTAGAAGAAGTGCAGTCACTT-3’ | This work |
| BgHR3-Fwd | 5’-GATGAGCTGCTCTTAAAGGCGAT-3’ | This work |
| BgHR3-Rev | 5’-AGGTGACCGAACTCCACATCTC-3’ | This work |
| dsBgE75-Fwd | 5’-AATGAGTAGAGATGCGGTGCGGTT-3’ | This work |
| dsBgE75-Rev | 5’-TCAGCGTCGGACAGTCTTAGTGA-3’ | This work |
| dsBgEcR-Fwd | 5’-GACAAACTCCTCAGAGAAGATCAAA-3’ | This work |
| dsBgEcR-Rev | 5’-CACATGTGCCACCGCGTCATCA-3’ | This work |
| Broad(+)Fwd | 5’-CATCTGCTCAGATACAGAACCT-3’ | This work |
| Broad(-)Rev | 5’-CTTCAGCAGCTGGTTGTTGATGT-3’ | This work |
| Dm(+)Fwd | 5´-ATGCACATCACCGATCACAG-3 | ^1^ |
| Dm(-)Rev | 5´-TGGGCCATCTGGAACTGTAG-3 | ^1^ |
| EcR(+)Fwd | 5’-ACTCCAGCCACAGATTCAACCACA-3’ | ^2^ |
| EcR(-)Rev | 5’-CATGTATTCGCTGCTCGTACTGAC-3’ | ^2^ |
| Eip74EFA(+)Fwd | 5’- GTTGCCGGAACATTATGGATATA-3’ | ^3^ |
| Eip74EFA(-)Rev | 5’- GCCCTATGTCGGCTTGCT-3’ | ^3^ |
| Eip74EFB(+)Fwd | 5’-ATCGGCGGCCTACAAGAAG-3’ | ^3^ |
| Eip74EFB(-)Rev | 5’-TCGATTGCTTGACAATAGGAATTTC-3’ | ^3^ |
| Eip75A(+)Fwd | 5-AATTCGCGGCGTGATCGACTT-3’ | ^2^ |
| Eip75A(-)Rev | 5’-AGCAACTTGGCCAGGAACTCG-3’ | ^2^ |
| Ftz-f1(+)Fwd | 5’-TGCGAGTCCTGCAAGGGATTCTTCA-3’ | This work |
| Ftz-f1(-)Rev | 5’-GCTCGAACAGCCTCTAGCTTCATGC-3’ | This work |
| Hr46(+)Fwd | 5’-GCTCAAATTGAGATAATTCC-3’ | This work |
| Hr46(-)Rev | 5'-ACTGCCGTAGCCCACCTCGT-3’ | This work |
| InR(+)Fwd | 5’-TCGTCGTGCGGAAAATCATCGC-3’ | This work |
| InR(-)Rev | 5’-CACACCCGGACAAGCCGCAC-3’ | This work |
| Kr-h1(+)Fwd | 5’-AAAGTATTACGAACGCAGCACCCA-3’ | This work |
| Kr-h1(-)Rev | 5’-GTCGCACTTGAATTGCGGTTTGCT-3’ | This work |
| RpL32(+)Fwd | 5’-CCTTCCAGCTTCAAGATGACCATCC-3’ | This work |
| RpL32(-)Rev | 5’-ATCCGTAACCGATGTTGGGCATCAG-3’ | This work |
| Thor(+)Fwd | 5´-TCCTGGAGGCACCAAACTTATC-3´ | ^4^ |
| Thor(-)Rev | 5´-GGAGCCACGGAGATTCTTCA-3 | ^4^ |

1: Bg, *Blatella germanica* sequences. The other sequences correspond to *Drosophila melanogaster*.

2: ds, oligonucleotides used for dsRNA synthesis. The other oligonucleotides were used for Q-PCR analysis.

**References**

1 Daneshvar, K. *et al.* MicroRNA miR-308 regulates dMyc through a negative feedback loop in Drosophila. *Biol Open* **2**, 1-9, doi:10.1242/bio.20122725 (2013).

2 Johnston, D. M. *et al.* Ecdysone- and NO-mediated gene regulation by competing EcR/Usp and E75A nuclear receptors during Drosophila development. *Mol Cell* **44**, 51-61, doi:10.1016/j.molcel.2011.07.033 (2011).

3 Caldwell, P. E., Walkiewicz, M. & Stern, M. Ras activity in the Drosophila prothoracic gland regulates body size and developmental rate via ecdysone release. *Curr Biol* **15**, 1785-1795, doi:10.1016/j.cub.2005.09.011 (2005).

4 Demontis, F. & Perrimon, N. FOXO/4E-BP signaling in Drosophila muscles regulates organism-wide proteostasis during aging. *Cell* **143**, 813-825, doi:10.1016/j.cell.2010.10.007 (2010).
